# Tuberculosis of the scalp: the tubercle bacilli has not ceased to surprise us

**DOI:** 10.11604/pamj.2017.28.28.13131

**Published:** 2017-09-13

**Authors:** Adil Zegmout, Hicham Souhi

**Affiliations:** 1 Service de Pneumologie, Hôpital Militaire d'Instruction Mohamed V, Rabat, Maroc; 2 Service de Pneumologie, Hôpital Militaire d'Instruction Mohamed V, Rabat, Maroc

**Keywords:** Tuberculosis, scalp, tubercle bacilli

## Image in medicine

A 52-year-old men was referred with a 6-months history of a 3cm single skin lesion, erythematous, not pruriginous, with a verrucous surface, surmounted by scales, with circinated border. His history included weight loss of 8kg, but no other associated symptoms or contact with tuberculosis patients. Erythrocyte sedimentation rate (ESR) was slightly elevated at 82mm in the first hour. Mycological sampling was negative. Tuberculin skin test was positive (15mm) and anti-human immunodeficiency virus serology was negative. A skin biopsy was performed and sent for histopathology and culture. The skin biopsy showed inflammatory granuloma composed mostly of epithelioid cells and giant cells, consistent with tubercular granuloma. Mycobacteria were absent on Ziehl-Neelsen staining, tubercle bacilli were isolated and grown in culture from the lesion and confirmed a scalp localisation of tuberculosis. The scalp localization of tuberculosis very rare and not sufficiently described in the literature and demonstrates the multifaceted aspect of tuberculosis, which continues to yield surprises with its unusual clinical manifestations. Antitubercular chemotherapy consisting of isoniazid, rifampicin, ethambutol and pyrazinamide was started. Follow-up at the end of treatment at 6 months showed a clinical remission.

**Figure 1:**
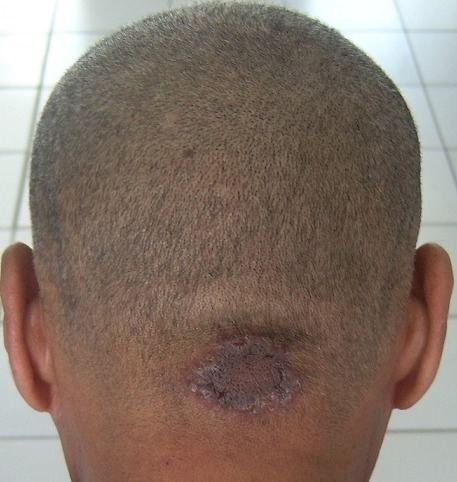
clinical photograph showing the lesion of scalp in occipital region

